# Biomarkers and Molecular Analysis to Improve Bloodstream Infection Diagnostics in an Emergency Care Unit

**DOI:** 10.1371/journal.pone.0087315

**Published:** 2014-01-27

**Authors:** Anne J. M. Loonen, Cornelis P. C. de Jager, Janna Tosserams, Ron Kusters, Mirrian Hilbink, Peter C. Wever, Adriaan J. C. van den Brule

**Affiliations:** 1 Jeroen Bosch Hospital, Laboratory of Molecular Diagnostics, 's-Hertogenbosch, The Netherlands; 2 Jeroen Bosch Hospital, Department of Intensive Care and Emergency Medicine, 's-Hertogenbosch, The Netherlands; 3 Jeroen Bosch Hospital, Department of Clinical Chemistry and Haematology, 's-Hertogenbosch, The Netherlands; 4 Jeroen Bosch Hospital, Jeroen Bosch Academy, 's-Hertogenbosch, The Netherlands; 5 Jeroen Bosch Hospital, Department of Medical Microbiology and Infection Control, 's-Hertogenbosch, The Netherlands; 6 Fontys University of Applied Sciences, Department of Medical Molecular Diagnostics, Eindhoven, The Netherlands; National Institute for Agriculture and Veterinary Research, IP (INIAV, I.P.), Portugal

## Abstract

Molecular pathogen detection from blood is still expensive and the exact clinical value remains to be determined. The use of biomarkers may assist in preselecting patients for immediate molecular testing besides blood culture. In this study, 140 patients with ≥ 2 SIRS criteria and clinical signs of infection presenting at the emergency department of our hospital were included. C-reactive protein (CRP), neutrophil-lymphocyte count ratio (NLCR), procalcitonin (PCT) and soluble urokinase plasminogen activator receptor (suPAR) levels were determined. One ml EDTA blood was obtained and selective pathogen DNA isolation was performed with MolYsis (Molzym). DNA samples were analysed for the presence of pathogens, using both the MagicPlex Sepsis Test (Seegene) and SepsiTest (Molzym), and results were compared to blood cultures. Fifteen patients had to be excluded from the study, leaving 125 patients for further analysis. Of the 125 patient samples analysed, 27 presented with positive blood cultures of which 7 were considered to be contaminants. suPAR, PCT, and NLCR values were significantly higher in patients with positive blood cultures compared to patients without (*p* < 0.001). Receiver operating characteristic curves of the 4 biomarkers for differentiating bacteremia from non-bacteremia showed the highest area under the curve (AUC) for PCT (0.806 (95% confidence interval 0.699–0.913)). NLCR, suPAR and CRP resulted in an AUC of 0.770, 0.793, and 0.485, respectively. When compared to blood cultures, the sensitivity, specificity, positive predictive value (PPV), and negative predictive value (NPV) for SepsiTest and MagicPlex Sepsis Test were 11%, 96%, 43%, 80%, and 37%, 77%, 30%, 82%, respectively. In conclusion, both molecular assays perform poorly when one ml whole blood is used from emergency care unit patients. NLCR is a cheap, fast, easy to determine, and rapidly available biomarker, and therefore seems most promising in differentiating BSI from non-BSI patients for subsequent pathogen identification using molecular diagnostics.

## Introduction

Bloodstream infection (BSI) is a potential life-threatening condition that requires early diagnosis and rapid pathogen identification to initiate correct antibiotic or antifungal therapy [1], [2], [3]. BSI patients frequently display characteristic symptoms of Systemic Inflammatory Response Syndrome (SIRS) [4]. In general, blood culture sets are collected when ≥ 2 SIRS symptoms are recognized and infection is suspected. Blood cultures are regarded as the “gold standard” for the detection of viable bacterial and fungal organisms from blood, but are time-consuming. Furthermore, the sensitivity of blood cultures decreases significantly when antibiotic therapy has been started before blood samples are taken [5], [6], or when fastidious or slow-growing pathogens need to be cultured.

Molecular assays may improve BSI diagnostics. Recently, several molecular assays became commercially available which can be used for pathogen detection from whole blood. SepsiTest (Molzym), a broad-range SYBR Green based real-time polymerase chain reaction (PCR) assay followed by sequencing, has been investigated in clinical studies and is considered a valuable tool in addition to blood cultures [7], [8]. Compared to blood cultures, the diagnostic sensitivity and specificity of the SepsiTest PCR were described to be 87.0 and 85.8%, respectively [8]. MagicPlex Sepsis Test (Seegene) screens for 90 pathogens and 3 resistance markers (mecA, vanA, vanB). Subsequently, 27 pathogens can be identified to the species level. Recently, MagicPlex Sepsis Test has been investigated for rapid detection of invasive candidiasis in pediatric patients, and was shown to have a sensitivity and specificity of 50% and 94%, respectively [9]. Currently no literature is available on the performance of MagicPlex Sepsis Test for detection of BSI in adults.

Several limitations of molecular assays currently exist. First, they require special pathogen DNA enrichment to detect the low number of pathogens present in whole blood samples. Second, technical expertise is required to perform the tests. Third, the clinical value of molecular assays remains to be elucidated. And finally, the DNA tests available are still expensive. These restrictions prevent molecular assays to become the next “gold standard” for diagnosis of BSI as it is difficult and costly to implement them in daily laboratory practise. Biomarkers can be used to preselect suspected BSI patients for additional DNA based assays.

Several biomarkers have been described as either being associated with the presence of BSI or suggested to have prognostic value for outcome of BSI. The most widely studied marker is C-reactive protein (CRP), which is an acute-phase protein released by the liver after the onset of inflammation. CRP is mostly used to assess the presence of infection and sepsis [10]. Procalcitonin (PCT) is the prohormone of calcitonin and was first reported as a marker of inflammation in 1993 [11]. Several studies have been published which investigated its clinical value in the diagnosis of bacterial infections, especially sepsis [12], [13]. Zahorec *et al*. were the first to propose to use the ratio of neutrophil and lymphocyte counts (neutrophil lymphocyte count ratio (NLCR)) as an additional marker of infection in clinical practice [14]. In patients with suspected community-acquired infection in an emergency care setting the NLCR proved to be a simple biomarker with discriminatory capacity in predicting bacteremia. Recently, it was shown that this marker can be used in the prediction of bacteremia in patients admitted to the emergency department [15].

The soluble form of the urokinase plasminogen activator receptor (suPAR) has gained growing interest because it is proposed as a predictor of disease severity and case fatality in patients with bacteremia [16]. suPAR plays a role in various immunological functions and is expressed on various cell types including neutrophils, lymphocytes, macrophages, endothelial cells and tumor cells [17]. The two biomarkers that have been most studied in patients with sepsis are CRP and PCT, both of which are described to be markedly elevated in patients with sepsis [18], [19].

In this study, we evaluated the ability of various biomarkers (CRP, PCT, suPAR, and NLCR) to predict BSI in patients with suspected community-acquired BSI upon admission to the emergency department (ED). Furthermore, the performances of two commercially available molecular assays were examined and compared to blood culture results.

## Materials and Methods

### Ethics statement

Individual patient consent was not obtained since all data used in this study were acquired retrospectively from the laboratory information system (LIS) without any additional blood sampling. The Internal Review Board of the Jeroen Bosch Hospital approved anonymous use of remnant whole blood, serum, and data retrieved from the LIS and waived the need for informed consent.

### Patients and microbiology

In this retrospective study, 140 patients presenting at the ED with ≥ 2 SIRS criteria as described by Bone *et al.* [4] were included during November-December 2011 and October-December 2012. Additional inclusion criteria were, (1) age above 18 years, (2) clinical suspicion of infection, (3) blood cultures ordered, (4) EDTA blood and serum drawn simultaneously with blood cultures, and (5) sufficient remnant EDTA blood and serum volume available for analysis. Fifteen patient samples were excluded from the study because of an alternative diagnosis without infection, leaving 125 patients for further analysis.

Blood cultures were drawn by the medical staff during the observation period in the ED. Routinely, two pairs of aerobic and anaerobic bottles were obtained and incubated for at least five days with a maximum of seven days (BacT/ALERT; bioMérieux, Marcy L'Etoile, France) or until positive. All isolates from positive blood cultures were identified at the species level by using standard microbiological procedures including MALDI-TOF mass spectrometry (Bruker Daltonics GmbH, Bremen, Germany). Positive blood cultures with Gram-positive skin bacteria, e.g. coagulase-negative staphylococci (CoNS) or infrequently isolated environmental bacteria were considered to be contaminants, and therefore excluded from statistical analysis.

After performing standard diagnostics, 1 ml remnant EDTA blood (per patient) was frozen (-80°C) in DNA free UMD tubes (Molzym, Bremen, Germany) until further processing. UMD tubes stabilize specimens, including whole blood, through avoidance of damage of pathogens by freeze-thaw effects. A minimum of 500 µl serum was frozen (-80°C) for retrospective determination of suPAR and procalcitonin levels.

### Biomarkers determination

CRP levels were measured with the Dimension Vista 1500 (Siemens Healthcare diagnostics). WBC counts were determined on a Sysmex XE-2100 hematology analyzer (Sysmex Corporation, Kobe, Japan). Neutrophil-Lymphocyte Count Ratio (NLCR) was determined by dividing the absolute neutrophil count by the absolute lymphocyte count. To additionally determine suPAR and PCT levels, serum was thawed on ice. suPAR levels were determined by using the suPARnostic ELISA kit (Virogates, Copenhagen, Denmark) according to the manufacturer’s instructions, and PCT levels were measured using the Cobas E411 (Roche Diagnostics).

### Molecular assays

UMD tubes containing 1 ml EDTA blood were thawed on ice. Subsequently, the entire sample (UMD storage buffer with 1 ml whole blood) was used for pathogen DNA isolation with the semi-automated MolYsis method (Molzym, Bremen, Germany) according to manufacturer’s guidelines (buffer volumes were adapted), except that whole blood samples have not been extracted in duplicate. Pathogen DNA was stored at –20°C until further processing.

The obtained pathogen DNA was analysed with two molecular assays (1) SepsiTest (Molzym, Bremen, Germany), and (2) MagicPlex Sepsis Test (Seegene, Seoul, Korea). Both molecular assays were used as described in the manufacturers’ manuals and can be used in combination with MolYsis pathogen DNA isolation.

SepsiTest is a broad-spectrum real-time PCR test using SYBR Green followed by sequencing of the positive samples. This test is able to detect more than 345 species within one working day (8 hours, including pathogen DNA isolation). Results were considered positive if sequencing was successful.

MagicPlex Sepsis Test is a real-time PCR test that screens for pathogens as well as for methicillin (mecA) and vancomycin (vanA and vanB) resistance at once. After creation of an amplicon bank via normal PCR, screening for more than 90 pathogens to the genus level and resistance markers is performed. Results are available within 5 hours (including pathogen DNA isolation). Subsequent selective identification of pathogens (only 27 pathogens can be identified to the species level) is possible within an additional 30 minutes. The 21 bacterial pathogens that can be identified to the species level are: *Pseudomonas aeruginosa, Acinetobacter baumannii, Stenotrophomonas maltophilia, Serratia marcescens, Bacillus fragilis, Salmonella typhi, Klebsiella pneumoniae, Escherichia coli, Klebsiella oxytoca, Enterobacter cloacae, Proteus mirabilis, Enterobacter aerogenes, Staphylococcus aureus, Staphylococcus epidermidis, Staphylococcus haemolyticus, Streptococcus agalactiae, Streptococcus pyogenes, Streptococcus pneumoniae, Enterococcus faecalis, Enterococcus faecium,* and *Enterococcus gallinarum*. In this study, the selective identification step for fungi was not performed (*Candida albicans, Candida tropicalis, Candida parapsilosis, Candida glabrata, Candida krusei,* and *Aspergillus fumigatus)*.

### Statistical analyses

Patients were divided in two groups, with or without positive blood cultures. The Chi-Square test was used to determine statistical differences based on age and gender distribution between the groups. With regard to the continuous variables, CRP, PCT, NLCR and suPAR, we firstly judged for fit to the normal distribution using stem-and-leaf plots and quantile-quantile plots. As our data did not follow a normal distribution, Mann-Whitney U tests were performed for comparison of variables in different groups. Receiver operating characteristics (ROC) curve analyses were performed for the single biomarkers and combinations in predicting blood culture positivity. The samples that were considered to be contaminated were labelled as “missing” and therefore not used for analyses. ROC curves displayed sensitivity versus 1-specificity such that area under the curves (AUC) varied from 0.5–1.0, with higher values indicating increased discriminatory ability. A *p*-value of less than 0.05 was considered statistically significant. Statistical analyses were performed using SPSS (Version 19.0. Armonk, NY: IBM Corp).

## Results

### Patients and microbiology

Of the 125 patient samples analysed, 27 patients (21.6%) presented with positive blood cultures (Table 1). In ten patients with positive blood cultures, *E. coli* was identified, in six patients *Staphylococcus* spp. including one *S. aureus* isolate, and in three patients *Streptococcus* spp. More Gram-negative bacteria were recovered (16/27, 59%) as compared to Gram-positive bacteria (11/27, 41%). All positive culture sets grew bacterial species, and no fungi were detected. Seven out of the 27 blood culture isolates were considered to contain contaminants of which five belonged to the group of CoNS. When excluding contaminants from the total results, 20/125 patients presented with clinically relevant positive blood cultures (16%).

**Table 1:** Microorganisms grown from positive blood cultures.

Gram-positive bacteria	Gram-negative bacteria
*Clostridium paraputrificum*	* *Brevundimonas diminuta*
* *Propionibacterium* sp.	*Enterobacter cloacae*
*Staphylococcus aureus*	*Escherichia coli* (10)
* *Staphylococcus capitis* (2)	*Klebsiella pneumoniae*
* *Staphylococcus epidermidis*	*Proteus mirabilis*
* *Staphylococcus hominis* (2)	*Proteus vulgaris*
*Streptococcus gallolyticus*	*Salmonella group C*
*Streptococcus pneumoniae*	
Viridans streptococci	
**11 (41%)**	**16 (59%)**

* Considered as contaminant. Number between () indicates number of cultures positive with this pathogen.

Relevant demographic data are depicted in Table 2. No significant difference was found when comparing gender distribution in both groups (with or without positive blood culture). However, patients with a positive blood culture were significantly older (68.9 ±17.3 years; mean ± SD) as compared to patients with a negative blood culture (60.4±18.0 years) (*p  = * 0.018).

**Table 2:** Data of study population and results for C-reactive protein (CRP), procalcitonin (PCT), soluble urokinase plasminogen activator receptor (suPAR), and neutrophil-lymphocyte count ratio (NLCR).

	Positive Blood Culture	Negative Blood Culture	*p*-value	AUC
Age (years) mean ±SD	68.9 (±17.3)	60.4 (±18.0)	0.018	
Gender	17 Male/10 Female	57 Male/41 Female	0.653	
CRP mean (± SD) (mg/L)	105 (±105)	119 (±110)	0.886	0.485
Median (range)	88 (8–371)	93 (1–490)		
PCT mean (± SD) (ng/mL)	11.1 (±25.2)	2.0 (±10.3)	< 0.001	0.806
Median (range)	1.0 (0.039–100)	0.2 (0.02–100)		
suPAR mean (± SD) (ng/mL)	10.0 (±6.2)	6.2 (±3.4)	< 0.001	0.793
Median (range)	8.7 (2.9–26.1)	5.5 (2.1–29.6)		
NLCR mean (± SD)	23.0 (±15.0)	12.2 (±9.1)	< 0.001	0.770
Median (range)	18.4 (7.1–56.5)	9.9 (0.89–44.1)		

**Figure 1:**
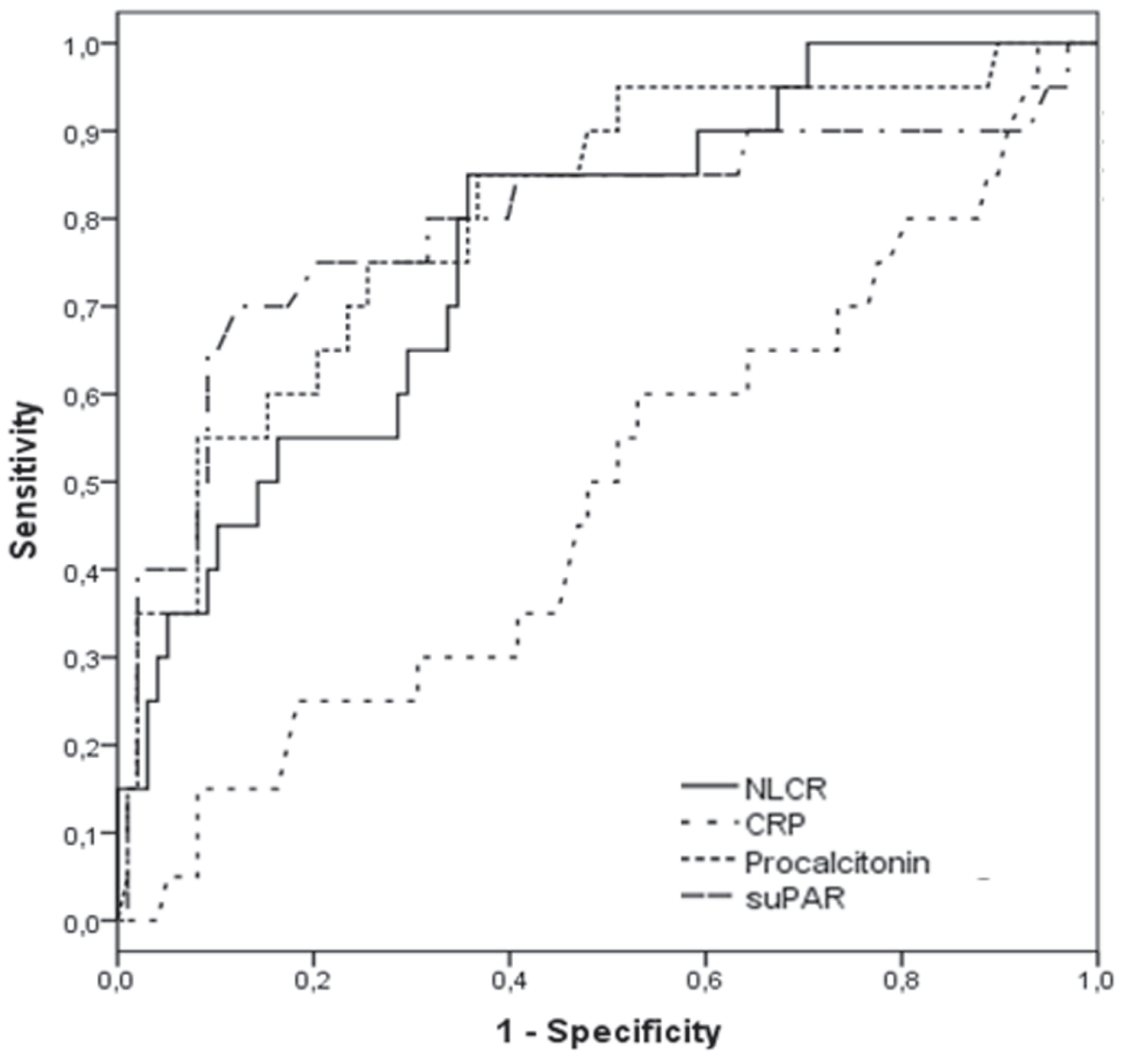
Receiver operating characteristic curves of four biomarkers for differentiating bacteremia from non-bacteremia. C-reactive protein (CRP), neutrophil-lymphocyte count ratio (NLCR), procalcitonin and soluble urokinase plasminogen activator receptor (suPAR) are compared in respect to prediction of positive blood culture.

Median and range, mean and standard deviation (SD) are displayed. *p-*value less than 0.05 is statistically significant (Chi-Square and Mann-Whitney *U* test), area under curve (AUC) is linked to Figure 1. The 7 patients with contaminated blood cultures were excluded from analyses.

### Biomarkers

Mean CRP levels between both groups (positive and negative blood culture) were similar (105±105 versus 119±110 mg/L, *p*  =  0.886) (Table 2). PCT levels were significantly different (*p* < 0.001) between patients with and without positive blood cultures (11.1±25.2 versus 2.0±10.3 ng/mL). The NLCR was also significantly different in both groups, showing a mean of 23.0±15.0 in patients with positive blood cultures. In the group with negative cultures a mean of 12.2±9.1 was found (*p* <0.001). A mean suPAR level of 10.0±6.2 ng/mL was found in the patient group having blood culture proven BSI as compared to a level of 6.2±3.4 ng/mL in the patient group with negative blood cultures (*p* < 0.001).

In our study population, 4 patients died as a result of sepsis complications. Blood culture results, NLCR, CRP, PCT, and suPAR levels in these patients were as follows: patient I) *K. pneumoniae,* 48.0, 228.0 mg/L, 100.0 ng/mL, 21.6 ng/mL, patient II) *S. epidermidis* (considered contaminant), 8.9, 165.0 mg/L, 1.04 ng/mL, 4.0 ng/mL, patient III) *S. gallolyticus,* 22.3, 76.0 mg/L, 0.255 ng/mL, 16 ng/mL, and patient IV) negative blood culture, 38.3, 42.0 mg/L, 0.065 ng/mL, 37.0 ng/mL.

ROC curve analysis showed that PCT had the highest area under the curve (AUC) for differentiating patients with blood culture proven BSI from patients without: 0.806 (95% confidence interval (CI) 0.699–0.913) (Figure 1). AUC values for the other biomarkers were as follows: suPAR 0.793 (95% CI 0.660–0.926), NLCR 0.770 (95% CI 0.662–0.879), and CRP 0.485 (95% CI 0.344–0.626). Regarding combinations of biomarkers, combining NLCR and suPAR was most promising and resulted in an AUC of 0.815.

Sensitivity, specificity, positive predictive value (PPV) and negative predictive value (NPV) for predicting blood culture proven BSI for PCT, NLCR and suPAR and combinations are depicted in Table 3. A 100% sensitivity and NPV were obtained when combining NLCR cut-off ≥ 10 with suPAR cut-off ≥ 6.2 ng/mL. However, specificity and PPV were low, i.e. 27% and 22%, respectively. By using only NLCR with a cut-off ≥ 10, sensitivity, specificity, PPV, and NPV were 85%, 51%, 26%, and 94%, respectively.

**Table 3:** Comparison of performance characteristics of the biomarkers and combinations in predicting bacteremia using different cut-off values.

	Sensitivity	Specificity	PPV	NPV
NLCR ≥ 10	85	51	26	94
NLCR ≥ 12	70	65	29	91
suPAR ≥ 6.2 ng/mL	85	58	29	95
suPAR ≥ 7.5 ng/mL	80	77	42	95
PCT ≥ 2	55	86	44	90
NLCR ≥ 10 and/or suPAR ≥ 6.2 ng/mL	100	27	22	100
NLCR ≥ 10 and/or PCT ≥ 2 ng/mL	95	49	28	98
NLCR ≥ 12 and/or suPAR ≥ 7.5 ng/mL	90	48	26	96
NLCR ≥ 12 and/or PCT ≥ 2 ng/mL	90	61	32	97

Abbreviations, PPV: positive predictive value, NPV: negative predictive value, NLCR: neutrophil-lymphocyte count ratio, suPAR: soluble urokinase plasminogen activator receptor, PCT: procalcitonin. The 7 patients with contaminated blood cultures were excluded from analyses.

Additionally, we analysed the efficiency of the biomarkers in predicting PCR and/or blood culture positivity (combined gold standard). On average the AUCs became lower, but CRP still remained <0.5, while the other three biomarkers (NLCR, suPAR and PCT) remained in close proximity of each other with AUCs between 0.70 and 0.73.

### Molecular assays

In Table 4 the results of the molecular assays are depicted and compared to the blood culture results. When using SepsiTest, only three EDTA blood samples were found positive, *S. gallolyticus, S. pneumoniae,* and *K. pneumoniae* were correctly identified as found in blood cultures. In contrast, MagicPlex Sepsis Test resulted in 12 positive PCR samples out of 27 blood culture positives. Detection of *S. aureus, E. coli* (6/10), *E. cloacae,* and *P. mirabilis* by MagicPlex Sepsis Test corresponded with blood culture results. The blood sample of one patient with a *S. capitis* blood culture isolate was only positive for *mecA* gene by MagicPlex Sepsis Test but not for *Staphylococcus* spp. MagicPlex Sepsis Test detected *K. pneumoniae* in the blood sample of one patient with a *S. hominis* blood culture isolate, while *S. epidermidis* was identified in blood from a patient with *S. gallolyticus* BSI.

**Table 4:** Overview of the results obtained with the molecular assays (SepsiTest and MagicPlex Sepsis Test) in comparison to blood culture results.

Blood Cultures	MolYsis + SepsiTest	MolYsis + MagicPlex Sepsis Test
Positive Culture (27)	Positive PCR/Sequencing (3)	Positive PCR (12)
*Streptococcus gallolyticus*	*S. gallolyticus*	*Staphylococcus epidermidis*
*Streptococcus pneumoniae*	*S. pneumoniae*	Negative
Viridans *Streptococcus* sp.	Negative	Negative
*Staphylococcus aureus*	Negative	*S. aureus*
*Clostridium paraputrificum*	Negative	Negative (not in kit)
*Escherichia coli* (10)	Negative (10)	*E. coli* (5), mix *S. aureus/E. coli* (1), negative (4)
*Enterobacter cloacae*	Negative	*E. cloacae*
*Klebsiella pneumoniae*	*K. pneumoniae*	Negative
*Proteus mirabilis*	Negative	*P. mirabilis*
*Proteus vulgaris*	Negative	Negative
*Salmonella* spp.	Negative	Negative
Contaminants (7)	Negative (7)	Negative (5), *K. pneumoniae* (culture *Staphylococcus hominis*), mecA (culture *Staphylococcus capitis*)
Additional PCR positives	4	23

SepsiTest resulted in four additional PCR positives blood samples from blood culture negative patients, i.e. *Corynebacterium tuberculostearicum*, *Shigella sonnei/flexneri*, *Malassezia* sp., and *Cryptococcus* sp. MagicPlex Sepsis Test resulted in an additional 23 PCR positive blood samples from culture negative patients: *S. epidermidis* (13), other CoNS (not in identification kit) (2), *S. pneumoniae* (2), fungi (2), *P. aeruginosa* (2), *A. baumannii* (1), and *K. oxytoca* (1). Cultures of other samples than blood (i.e urine, throat swabs, sputa, feces) could not explain our additional PCR positives.

In this study, the sensitivity, specificity, PPV, and NPV for SepsiTest and MagicPlex Sepsis Test (using blood culture as “gold standard”) were 11%, 96%, 43%, 80%, and 37%, 77%, 30%, 82%, respectively. Negative and positive controls, included in each assay, showed adequate results indicating that each assay was performed correctly.

## Discussion

Early identification of the pathogen causing BSI is essential for its adequate treatment, and it has been shown that when this treatment is initiated rapidly this will decrease BSI related mortality [20], [21]. Several tools are available which can be used and combined for optimal patient care: blood cultures, serum biomarkers, and potentially molecular assays for whole blood analysis.

Here, we studied the potential discriminating power of several biomarkers in the prediction of BSI in patients with SIRS and community-acquired infections presenting at the emergency department. In addition the performance of molecular assays was compared to the diagnostic yield of standard blood cultures. PCT, NLCR and suPAR are able to differentiate SIRS patients with and without blood culture proven bacteremia. The additional value of molecular assays in predicting BSI was low.

In this study, all patients fulfilled ≥ 2 SIRS criteria and showed signs of infection, while only 16% presented with clinically relevant positive blood cultures (20/125). When including the blood culture contaminants, 22% of the patients had positive blood cultures (27/125). Other studies describe similar numbers. In a study analysing 104 patients of a surgical intensive care unit (ICU), Lodes *et al.* found that 20% of the drawn blood culture sets became positive [22]. Fitting *et al.* found 23% positive blood cultures in their ICU patient population [23]. However, Hoenigl *et al.* reported a positivity rate of 41% in a patient population from the emergency department [24]. Hoenigl *et al.* and Lodes *et al.* both used the BACTEC blood culture system as compared to the BacT/ALERT system used in this study. It has been described that BACTEC media has faster time to detection and increased bacterial recovery over the BacT/ALERT media [25]. Besides differences in culture systems, only two blood culture sets were drawn in this study, while Hoenigl *et al.* collected three pairs of blood cultures per patient [24]. It remains difficult, however, to directly compare these study results as patient characteristics including disease severity might also differ besides culture methods.

PCT has been described to strongly correlate with the extent and severity of bacterial infections [26]. PCT is most frequently used in the management of infection and sepsis. Mencacci *et al.* have investigated if PCT serum levels could predict a positive PCR result (Septi*FAST*, Roche) [27]. They found that PCT (cut-off value ≥ 0.37 ng/ml) could be used in an unselected population of patients with fever and suspected sepsis to predict Septi*FAST* PCR results. We have shown that PCT, as compared to the other biomarkers in this study, has the highest specificity in predicting bacteremia in SIRS patients. However, as PCT is an expensive biomarker, pre-screening of SIRS patients for further DNA analysis using PCT might be less cost-effective.

The use of suPAR levels at the emergency department has mostly been described in relation to categorizing patients according to their disease severity [28]. In this study, significant differences were found for suPAR levels between SIRS patients with and without positive blood cultures. However, only 2/4 patients who died had high suPAR levels (>16 ng/mL). The suPAR levels of these patients were found between 4.4 – 26 ng/mL. More studies, investigating suPAR levels in patients from the emergency department, need to be performed to clarify the usefulness of this relatively new biomarker.

De Jager *et al*. have described the NLCR in relation to predicting bacteremia at the emergency department [15]. NLCR (cut-off ≥10) was shown to have a higher prognostic accuracy as compared to other biomarkers. In this study, prediction of community-acquired bacteremia using a NLCR cut-off ≥10 resulted in a sensitivity, specificity, PPV, and NPV of 85%, 51%, 26%, and 94%, respectively. Based on NLCR (cut-off ≥10), 65/118 patients in our study would have been selected for molecular analysis. A higher cut-off value, i.e. ≥12, results in decreased sensitivity but increased specificity. However, it is important to avoid SIRS patients to be falsely considered as negative BSI cases. For pre-screening purposes high sensitivity is preferred.

In this study, we investigated the performances of two molecular assays as compared to blood cultures. The sensitivities and specificities for SepsiTest were 11% and 96% compared to 37% and 77% for MagicPlex Sepsis Test. In this study, MagicPlex Sepsis Test and blood cultures showed similar results in 72% of the samples (both positive and negative samples). No reports were found describing the performance MagicPlex Sepsis Test in adult SIRS patients. Therefore, we can only compare the obtained SepsiTest results to other published studies. Kuhn *et al.* described a sensitivity of 85% for SepsiTest in patients with endocarditis [7]. Wellinghausen *et al*. describe a concordance of 86% for SepsiTest PCR and blood cultures in ICU patients with SIRS or sepsis [8]. Although, in this study, a concordance of 82% was found between SepsiTest and blood cultures for patients presenting with SIRS symptoms at the emergency department, the sensitivity compared to the blood culture gold standard was only 11%. Different patient populations were used (ICU versus emergency department), and this might explain the difference in patient-related sensitivity (85% and 82.4% versus 11% in this study). The performance of molecular assays is mostly investigated in ICU patient populations [8], [23], [29]. The prevalence of sepsis in ICU patients is very high, and most patients have clinically or microbiologically documented infection [30]. We speculate that the ICU patient population might suffer from higher bacterial loads which can be more easily detected from one ml whole blood. It would be interesting to study bacterial loads in different patient populations.

Four patients from our study population died, and 3 of those patients had positive blood cultures (*Klebsiella pneumoniae, Streptococcus gallolyticus* and *Staphylococcus epidermidis*). SepsiTest correctly identified *K. pneumoniae* and *S. gallolyticus,* whereas MagicPlex Sepsis Test did not identify these pathogens from whole blood. As these patients died, this might indicate that these patients were more critically ill and suffered from higher bacterial loads, as has been described by Peters *et al.* [31]. It is unknown why MagicPlex Sepsis Test did not show positive PCR signals in the whole blood samples of these specific patients.

SepsiTest resulted in four PCR positive samples from blood culture negative patients. Three out of these four can be considered contaminants as these can be found on skin (*Corynebacterium tuberculostearicum*, *Malassezia* sp., and *Cryptococcus* sp). However, *Shigella sonnei/flexneri* is a clinically significant pathogen that was not detected with blood culture. MagicPlex Sepsis Test resulted in an additional 23 PCR positive samples in blood culture negative patients. Based on the clinical presentation of the patients, the professional opinion of our clinical microbiologists and the local historical epidemiological evidence, the CoNS species (15) were considered to be contaminants. However, *S. pneumoniae* (2), fungi (2), *P. aeruginosa* (2), *A. baumannii* (1), and *K. oxytoca* (1) are clinical relevant pathogens. Prospective studies are needed to investigate the clinical value of additional positive samples using molecular diagnostics.

Molecular-based technologies are emerging as promising tools, in addition to blood cultures, for rapid identification of the etiological agents of BSI [32], [33]. However, several limitations exist (i.e. costs and the need for special equipment) which negatively affect the implementation of these techniques for routine laboratory diagnostics [34]. A limitation of this study is the use of only one ml residual whole blood for molecular analysis. It has been shown that detection rates obtained using molecular assays are higher when 5 ml whole blood is used as compared to only one ml [35]. However, in this study residual whole blood, which was left after performing standard diagnostic tests, was used and only one ml whole blood could be processed and not 5 ml. More detailed investigation is necessary to select the best molecular assay available today. Furthermore, only prospective studies should be performed, using at least 5 ml (or more) fresh whole blood from a larger cohort of suspected BSI patients. This should allow more optimal detection of pathogens from whole blood. Besides that, cost effectiveness analysis is needed to study the effect of implementation of a molecular assay on patient samples preselected, for instance based on NLCR results, in addition to blood cultures.

In summary, of all the biomarkers studied, PCT, suPAR and NLCR are suitable to differentiate SIRS patients with and without positive blood cultures. NLCR is a rapidly available, cheap, and easy to determine biomarker. Therefore, NLCR is a promising biomarker to preselect suspected BSI patients for molecular analysis besides blood culture. Unfortunately, the molecular assays available to date are not yet suitable for analysis of one ml remnant whole blood samples from patients at the emergency care unit. In addition, the clinical significance of DNA positivity in blood samples which remain culture negative needs further investigation. Currently, we do not have enough results available to implement a laboratorial testing algorithm in which BSI-suspected patients are selected for a molecular assay based on i.e. NLCR results. We hope that future prospective studies can guide towards such an algorithm.
